# Molecular characterization of homogentisate phytyltransferase and methylphytylbenzoquinol methyltransferase genes from olive fruit with regard to the tocopherol content and the response to abiotic stresses

**DOI:** 10.3389/fpls.2025.1526815

**Published:** 2025-03-03

**Authors:** Isabel Narváez, M. Luisa Hernández, M. Dolores Sicardo, David Velázquez-Palmero, Wenceslao Moreda, José M. Martínez-Rivas

**Affiliations:** Departamento de Bioquímica y Biología Molecular de Productos Vegetales, Instituto de la Grasa (IG), Consejo Superior de Investigaciones Científicas (CSIC), Seville, Spain

**Keywords:** *Olea europaea*, olive, vitamin E, HPT, MPBQ MT, gene expression, abiotic stresses

## Abstract

Two cDNA sequences, named *OepHPT* and *OepMPBQ MT*, encoding homogentisate phytyltransferase (HPT) and methylphytylbenzoquinol methyltransferase (MPBQ MT), respectively, have been cloned from olive (*Olea europaea* cv. Picual). Sequence analysis displayed the distinguishing characteristics typical of the HPT and MPBQ MT families and along with phylogenetic analysis indicated that they code for homogentisate phytyltransferase and methylphytylbenzoquinol methyltransferase enzymes, respectively. Transcriptional analysis in distinct olive tissues indicated that expression levels of *HPT* and *MPBQ MT* genes are spatially and temporally regulated in a cultivar-dependent manner and together with tocopherol analysis pointed out that both genes participate in the biosynthesis of the tocopherols present in olive mesocarp. These data also suggest that in olive mesocarp, HPT but not MPBQ MT could be implicated in the transcriptional regulation of the tocopherol biosynthetic pathway. In addition, *HPT* and *MPBQ MT* transcript levels are regulated by water status, temperature, light, and wounding in the olive fruit mesocarp, suggesting that both genes could be implicated in the abiotic stress response. Overall, this research constitutes a significant advance to elucidate the factors that regulate the tocopherol biosynthesis in olive fruit to obtain virgin olive oils with enhanced α-tocopherol content.

## Introduction

In nature, four tocopherols and four tocotrienols have been described, constituting a group of lipid-soluble antioxidants named tocochromanols that belong to the group of vitamin E compounds (Fritsche et al., 2017). The basic structure of tocochromanols is simple, comprising a polar chromanol ring and a lipophilic polyprenil side chain, which is saturated in tocopherols and 3-fold unsaturated in tocotrienols (Falk and Munné-Bosch, 2010). The number of methyl groups in the chromanol ring determines the four α- (three-methyl groups), β- and γ- (two-methyl groups), and δ- (one-methyl group) tocopherol and tocotrienol subforms (Mène-Saffrané, 2018). Tocopherols are found in all photosynthetic organisms, whereas tocotrienols are only present in certain plant groups (Falk and Munné-Bosch, 2010).

In higher plants, the content and composition of tocopherols differ considerably in distinct tissues, being generally present in the green parts (Horvath et al., 2006). Commonly, seeds of many plant species accumulate γ-tocopherol, while leaves are rich in α-tocopherol (Hussain et al., 2013), with some important exceptions such as sunflower where the main tocopherol form in the seed is α-tocopherol (Velasco et al., 2002). α-Tocopherol is localized in the chloroplasts, specifically in the inner envelope and thylakoid membranes, and in some cases in plastoglobuli (Hussain et al., 2013).

Tocopherol biosynthesis takes place in the plastids of photosynthetic organisms. The first reaction of the pathway consists in the condensation of the polar aromatic head group homogentisic acid (HGA) with the polyprenyl side chain phytyl diphosphate (PDP), which is catalyzed by the enzyme homogentisate phytyltransferase (HPT), producing 2-methyl-6-phytyl-1,4-benzoquinol (MPBQ) (Lushchak and Semchuk, 2012; Mène-Saffrané, 2018). Regarding the origin of both substrates, the formation of HGA is derived from the shikimate pathway and is catalyzed by the *p*-hydroxyphenylpyruvate dioxygenase, whereas PDP is originated from the methylerytrithol phosphate pathway and synthesized by the geranylgeranyl reductase from geranylgeranyl diphosphate, as well as from the recycling of free phytol derived from chlorophyll degradation catalyzed sequentially by two phytol kinases (Pellaud and Mène-Saffrané, 2017; Niu et al., 2022). The reaction product MPBQ can be methylated via MPBQ methyltransferase (MPBQ MT) to 2,3-dimethyl-6-phytyl-1,4-benzoquinol (DMPBQ). MPBQ and DMPBQ are then further cyclized by the tocopherol cyclase (TC) to produce δ- and γ-tocopherols, respectively. Finally, the enzyme γ-tocopherol methyltransferase (γ-TMT) catalyzes the methylation of δ- and γ-tocopherols into β- and α-tocopherols, respectively (Fritsche et al., 2017).

HPT catalyzes the first committed step of tocopherol biosynthesis. Arabidopsis plants lacking HPT are completely deficient in all tocopherols and pathway intermediates in both, seeds and leaves, indicating that this is a limiting step in tocopherol biosynthesis (Sattler et al., 2004). The genes encoding HPT, also named *VTE2*, were initially cloned from Arabidopsis and *Synechocystis* (Collakova and DellaPenna, 2001; Schledz et al., 2001). Overexpression of *AtHPT* in Arabidopsis plants resulted in a 2-fold increment in total tocopherol level in seeds (Savidge et al., 2002; Collakova and DellaPenna, 2003a). In the same way, overexpression of *AtHPT* in rapeseed plants increased the total tocopherol content in the transgenic seeds (Lassner et al., 2001). In contrast, overexpression of *HPT* genes in soybean did not significantly enhance the tocopherol content in the seeds (Karunanandaa et al., 2005). These data suggest that HPT activity is at least partially limiting in seeds. Interestingly, only a minor increment was observed when the seed-specific expression of the *Synechocystis HPT* gene was carried out in rapeseed, indicating species-specific differences in the regulation of tocopherol biosynthesis (Karunanandaa et al., 2005). In leaves, the constitutive overexpression of the *HPT* gene brought about a considerable increase in the total tocopherol content in Arabidopsis (Collakova and DellaPenna, 2003b; Li et al., 2010), tomato (Seo et al., 2011; Lu et al., 2013), and lettuce (Lee et al., 2007). Overall, these results indicate that HPT clearly limits leaf tocopherol synthesis, whereas it is not a major bottleneck in tocopherol metabolism in seeds (Mène-Saffrané and Pellaud, 2017).

The MPBQ MT enzyme catalyzes the step of the tocopherol biosynthetic pathway that controls the ratio between α- and γ-tocopherols with respect to β- and δ-tocopherols, competing for the same MPBQ substrate with the TC enzyme. Additionally, the MPBQ MT enzyme is also involved in the methylation of 2-methyl-6-solanyl-1,4-benzoquinone (MSBQ) to produce plastoquinol-9 in the synthesis of plastoquinones (Lushchak and Semchuk, 2012). The first *MPBQ MT* gene, also called *VTE3*, was isolated from *Synechocystis* because of its homology to γ-TMT (Shintani et al., 2002). Arabidopsis *vte3-2* mutant lines, which have a complete gene disruption, were deficient in α- and γ-tocopherols, as well as plastoquinones, but accumulating β- and δ-tocopherols (Cheng et al., 2003; Motohashi et al., 2003). Overexpression of *AtMPBQ MT* in Arabidopsis and soybean seeds did not have a substantial increase in total tocopherol amounts, but highly altered tocopherol composition decreasing β- and δ-tocopherols, with a corresponding increment in α- and γ-tocopherols (Van Eenennaam et al., 2003; Li et al., 2010).

In photosynthetic organisms, the main physiological role of tocopherols has been associated with their non-enzymatic antioxidant activity that protects lipids from oxidation by scavenging lipid peroxy radicals and quenching singlet molecular oxygen during stress conditions (Munné-Bosch and Alegre, 2002). More recently, several studies suggest that tocopherols are essential for plant development and are involved in several functions such as the plant response to environmental stresses, the maintenance of membrane stability, transcript regulation, and intracellular signaling (Fritsche et al., 2017; Muñoz and Munné-Bosch, 2019; Ma et al., 2020; Niu et al., 2022; Mesa and Munné-Bosch, 2023).

In mammalian systems, the role of vitamin E has been widely studied since its discovery in 1922. In addition to its essential role in animal reproduction, vitamin E has beneficial functions in human health (Galli et al., 2017). A sufficient uptake of vitamin E can help to prevent chronic diseases and neurological disorders, mainly those with an oxidative stress component such as cancer or atherosclerosis (Li et al., 2011; Qureshi et al., 2002). Among tocochromanols, the most biologically active form of vitamin E is α-tocopherol because it exhibits the highest affinity to the α-tocopherol transfer protein, a cytoplasmic protein that transports tocochromanols from the endosomal fraction of hepatocytes into the bloodstream of animals (Hosomi et al., 1997). Because humans cannot synthesize vitamin E, it is an essential part of our diet. It is commonly found in nuts, seeds, grains, and vegetable oils, but also in fruits and leafy vegetables (DellaPenna and Last, 2006). Wheat germ and sunflower oils are the best sources of α-tocopherol, while γ-tocopherol can be found in high concentrations in rapeseed, corn, and soybean oils (Brigelius-Flohe and Traber, 1999). Therefore, the enrichment in vitamin E content and the increase of α-tocopherol in vegetable oils are of particular interest in crop breeding (Fritsche et al., 2017).

Virgin olive oil (VOO) is mainly constituted of triacylglycerols esterified by fatty acids, which are the major components (≥98%), and a highly diverse mixture of chemical compounds called “minor compounds” (≤2%) (Aparicio and Harwood, 2013). Among the minor components present in the VOO are the tocopherols. Their total content ranges between 90 and 1400 mg/kg, with α-tocopherol representing more than 95% (Pérez et al., 2019). β- and γ-tocopherols are also present but only in minor amounts. Almost all the tocopherols present in VOO derive from the mesocarp of the olive fruit, with a minor contribution from the seed (Lavelli and Bondesan, 2005). In olive mesocarp, total tocopherols varied from 135 to 579 mg/kg (Mousavi et al., 2022). Tocopherol content and composition in VOO and olive fruit are affected by genetic and agronomic factors such as cultivar, fruit ripeness, and agroclimatic conditions (Beltrán et al., 2010; Mousavi et al., 2022). Unlike the phenolic compounds with secoiridoid structures, tocopherols do not suffer any relevant chemical or enzymatic transformation during the oil extraction process, being directly transferred from the olive fruit to the VOO. However, a certain loss of tocopherols is produced in the added water during the centrifugation step due to their amphipathic nature (Oliveras-López et al., 2008).

In recent years, the generation of novel olive cultivars producing VOO with improved functional quality, including the increase in vitamin E content, has been considered an important target of olive breeding programs (Pérez et al., 2019). To achieve that, the identification of molecular markers associated with high vitamin E content in VOO is needed. However, unlike oilseeds and leaves, molecular studies about tocopherol biosynthesis in oil fruits are very limited. In olive, no genes specific to the tocopherol biosynthetic pathway have been cloned and characterized to date, although their expression levels have been measured in the mesocarp of several Greek olive cultivars (Georgiadou et al., 2015; Georgiadou et al., 2016; Georgiadou et al., 2019). In the present work, we report the isolation and characterization of *HPT* and *MPBQ MT* genes in olive. Tocopherol and expression analysis during the development and ripening of olive fruit from different cultivars were performed to investigate their specific roles in tocopherol biosynthesis and their possible implication in the response to several environmental stresses in the olive mesocarp.

## Materials and methods

### Plant material and stress treatments

Olive (*Olea europaea* L.) trees (cv. Picual and Arbequina) were cultivated in the experimental orchard of Instituto de la Grasa, Seville (Spain), using drip irrigation and fertirrigation (irrigation with suitable fertilizers in the solution) from the time of full bloom to fruit ripening. Young drupes, developing seeds, and mesocarp tissue were collected from at least three different olive trees at different weeks after flowering (WAF) corresponding to distinct developmental stages of the olive fruit: green (9, 12, 16, and 19 WAF); yellowish (23 WAF); turning or veraison (28 and 31 WAF); and mature or fully ripen (35 WAF). Young leaves were harvested as well.

Olive trees (cv. Klon-14, Abou Kanani, Dokkar, and Piñonera) from the Worldwide Olive Germplasm Bank of Córdoba located at IFAPA (Alameda del Obispo, Córdoba, Spain) were grown by using standard culture practices. Mesocarp tissue from these cultivars was collected at distinct developmental stages of the olive fruit: green (20 WAF); yellowish (24 WAF); turning or veraison (27 WAF) and mature or fully ripen (31 WAF).

To study the effect of water deficit, mesocarp tissue at different WAF was collected at the Sanabria orchard, a commercial super high-density olive (cv. Arbequina) orchard near Seville, as previously described (Hernández et al., 2018). Full irrigation (FI) and two regulated deficit irrigation (RDI) treatments (60RDI and 30RDI) were applied.

Stress treatments were conducted as described by Hernández et al. (2019) using olive branches collected from different olive trees (cv. Picual and Arbequina) with around 100 olive fruit at the turning stage (28 WAF) and incubated in a growth chamber with a 12 h light/12 h dark cycle (light intensity of 300 µmol m^-2^ s^-1^.) at 25°C. These incubation parameters were considered the standard conditions since they mimic the physiological conditions of the tree. For stress treatments, standard conditions were changed according to the effect studied. To assess the influence of low and high temperatures, the branches were incubated at 15 or 35°C, respectively. To study the impact of the darkness, the light was turned off. For wounding experiments, the entire surface of the olive fruit was mechanically damaged affecting mesocarp tissue, with pressure at zero time using forceps with serrated tips. The zero time of each treatment was chosen 2 h after the beginning of the light period to preserve the natural photoperiod day/night of the olive fruit. The duration of stress exposure was 24 h. This short-term exposition was selected because longer times can cause tissue damage in the olive fruits, since they are not in the olive tree but in branches incubated in chambers.

In all cases, olive tissues were frozen in liquid nitrogen immediately after harvest and stored at -80°C.

### Isolation of homogentisate phytyltransferase and methylphytylbenzoquinol methyltransferase full-length cDNA clones

Candidate olive *HPT* and *MPBQ MT* sequences were identified in the olive transcriptome (Muñoz-Mérida et al., 2013) and the wild olive (var. sylvestris) genome (Unver et al., 2017) using the tblastn algorithm together with the amino acid sequences of Arabidopsis *HPT* and *MPBQ MT* genes (At2g18950 and At3g63410, respectively). Based on these two new sequences, specific pairs of primers for each gene were designed and used for PCR amplification with VELOCITY DNA polymerase (Bioline, Spain), which has proofreading activity. An aliquot of an olive Uni-ZAP XR cDNA library constructed with mRNA isolated from 13 WAF olive (cv. Picual) fruit (Haralampidis et al., 1998) was utilized as a DNA template. One fragment with the expected size was obtained in each reaction, subcloned into the vector pSpark^®^ I (Canvax, Spain), and sequenced in both directions. DNA sequencing was carried out by GATC Biotech (Germany).

The DNA sequence data were compiled and analyzed with the LASERGENE software package (DNAStar, Madison, WI). The multiple sequence alignments of olive HPT and MPBQ MT amino acid sequences were calculated using the ClustalX program and displayed with GeneDoc. Phylogenetic tree analysis was performed using the neighbor-joining method implemented in the Phylip package using Kimura’s correction for multiple substitutions and a 1000 bootstrap data set. TreeView was used to display the tree. The conserved domains in the deduced amino acid sequences were analyzed using the NCBI Conserved Domain Search (http://www.ncbi.nlm.nih.gov/structure/cdd/wrpsb.cgi) and Pfam software (https://pfam.xfam.org/). TMHMM analysis was carried out (http://www.cbs.dtu.dk/services/TMHMM/) and subcellular localization was predicted using three different programs: ProtComp 9.0 (http://www.softberry.com), WoLF PSORT (https://wolfpsort.hgc.jp/) and TargetP-2.0 (http://www.cbs.dtu.dk/services/TargetP/). Prediction of N-terminal chloroplast targeting peptides was performed using ChloroP 1.1 software (http://www.cbs.dtu.dk/services/ChloroP/).

### Total RNA isolation and cDNA synthesis

Total RNA isolation was carried out according to Hernández et al. (2005) using 1.5 g of frozen olive tissue. RNA quality verification, removal of contaminating DNA, and cDNA synthesis were performed as described by Hernández et al. (2009).

### Expression analysis of homogentisate phytyltransferase and methylphytylbenzoquinol methyltransferase genes

Gene expression levels of the olive *HPT* and *MPBQ MT* were analyzed by quantitative real-time PCR (qRT-PCR) using a CFX Connect real-time PCR System and iTaq Universal SYBR Green Supermix (BioRad, California, USA) as described by Hernández et al. (2019). Primers for gene-specific amplification of *OeHPT* and *OeMPBQ MT* were designed using the Primer3 program (http://bioinfo.ut.ee/primer3/) and the Gene Runner program (**Supplementary Table 1**). The housekeeping olive ubiquitin2 gene (*OeUBQ2*, AF429430) was used as an endogenous reference to normalize (Hernández et al., 2009). The qRT-PCR data were calibrated relative to the corresponding gene expression level at 12 WAF ‘Picual’ mesocarp for developmental studies, at 13 WAF ‘Arbequina’ mesocarp of FI treatment for water deficit study, and zero time for each stress treatment and cultivar, respectively, as calibrator. The relative expression level of each gene was calculated following the 2^-ΔΔCt^ method for relative quantification (Livak and Schmittgen, 2001). The data are presented as means ± SD of three biological replicates, each having two technical replicates per 96 well plate.

### Tocopherol analysis

Lyophilized mesocarp tissue (500 mg DW) was processed in a glass tube with 3 mL of hexane and the mixture was shaken for 15 min including 1 min vortexing. After centrifugation at 2000 rpm for 10 min at RT, the hexane phase was recovered, and the tissue was extracted twice. The hexane phases were mixed, and hexane was added to complete an exact volume of 10 mL in a graduated flask. An aliquot of the extracts was filtered and injected into an HPLC. The tocopherols were determined according to Abdallah et al. (2015), using a Supersphere Si60 Lichrocart 250-4 HPLC cartridge (Merck), and eluted with hexane:2-propanol (99:1) at a flow rate of 1 mL/min. A fluorescence detector (Shimadzu RF535) was used, setting the excitation and emission wavelengths at 290 and 330 nm, respectively. The injection volume of the sample was 20 µL. Peaks of α-, β-, γ- and δ-tocopherol were identified using a mix standard solution of all the four compounds. The quantitative evaluation was performed by external standardization using an α-tocopherol calibration curve. The data are presented as means ± SD of three biological replicates, each having three technical replicates.

## Results and discussion

### cDNA cloning and sequence analysis of olive homogentisate phytyltransferase and methylphytylbenzoquinol methyltransferase genes

**Figure 1:**
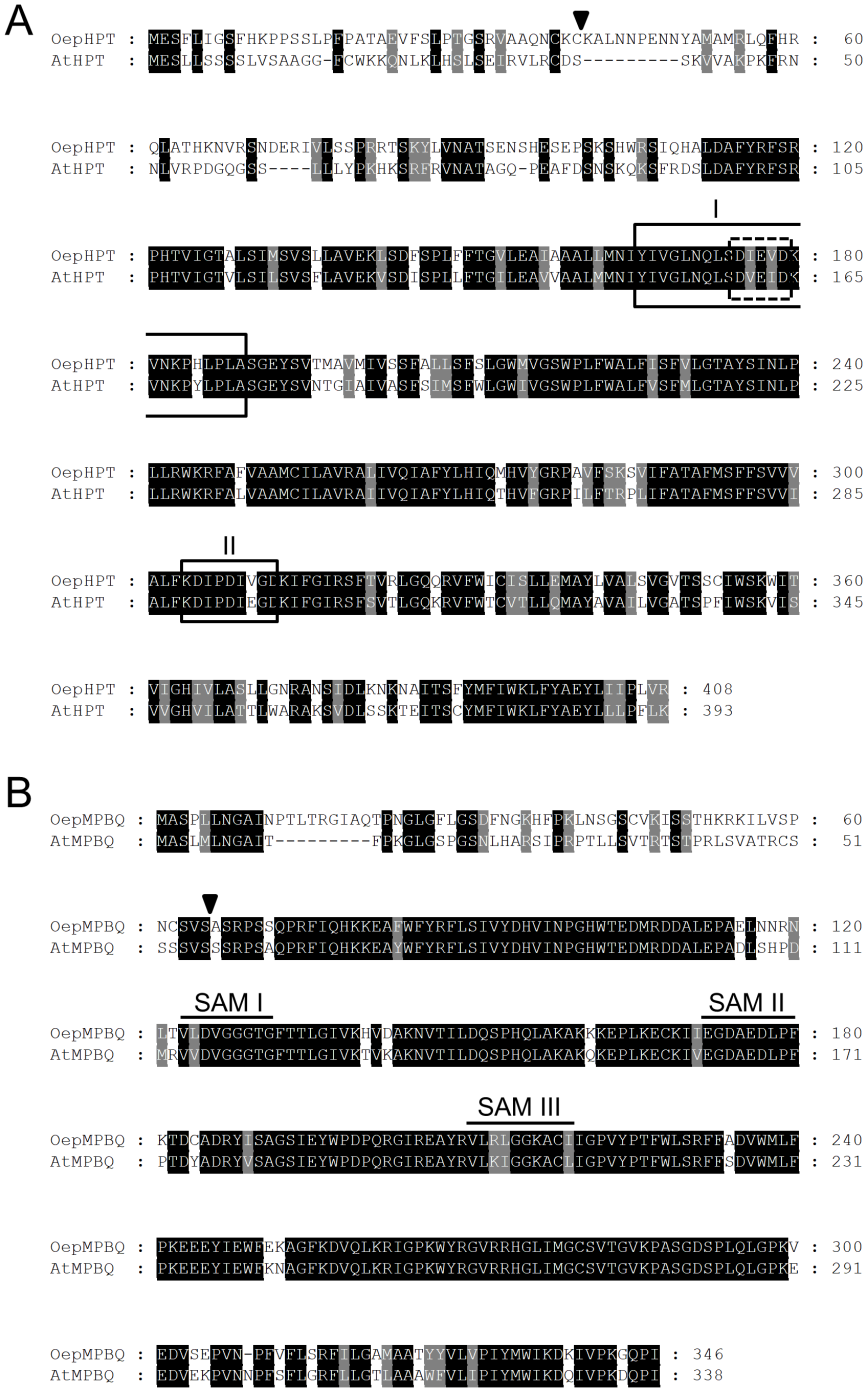
Comparison of the deduced amino acid sequences of olive *HPT* **(A)** and *MPBQ MT* **(B)** genes, with those from Arabidopsis. The sequences were aligned using the ClustalX program and displayed with GeneDoc. Identical and similar residues are shown on a background of black and grey, respectively. The putative cleavage sites of the chloroplast transit peptides are indicated by triangles. In the case of HPT, the prenyl-DP binding motif I and the divalent cation binding domain, both typical for polyprenyltransferases, are boxed with solid and dashed lines, respectively, and the Asp-rich motif II, which is involved in substrate binding is framed with a solid line. Regarding MPBQ MT, the three conserved domains SAM I, II, and III characteristics of *S*-adenosylmethionine-dependent methyltransferases are denoted by continuous lines. The cDNA sequences corresponding to *OepHPT* and *OepMPBQ MT* have been deposited in the GenBank/EMBL/DDBJ database with accession numbers PQ479102 and PQ479103, respectively. The accession numbers of AtHPT and AtMPBQ MT are AF324344 and CAB87794, respectively.

Two sequences were identified from the olive transcriptome (Muñoz-Mérida et al., 2013) and the olive (var. sylvestris) genome (Unver et al., 2017), which displayed an elevated degree of similarity to the Arabidopsis *HPT* and *MPBQ MT* genes (Collakova and DellaPenna, 2001; Cheng et al., 2003). Based on these sequences, specific primer pairs were designed and utilized for PCR amplification, along with an aliquot of an olive fruit (13 WAF) cDNA library (cv. Picual). Two full-length cDNA clones were isolated and named *OepHPT* and *OepMPBQ MT*, with sizes of 1296 and 1264 bp, respectively. They exhibited ORFs encoding predicted proteins of 408 and 346 amino acid residues, which correspond to calculated molecular masses of 45.7 and 38.8 kDa, respectively, and p*I* values of 9.6 for OepHPT and 9.1 for OepMPBQ MT. Alignment of the olive HPT deduced amino acid sequences with Arabidopsis HPT indicated that they displayed 60% identity (**Figure 1A**), while olive MPBQ MT shared 75% identity with Arabidopsis MPBQ MT (**Figure 1B**).

The Conserved Domain search revealed that the olive HPT protein belongs to the *PLNO2878* superfamily, with homogentisate phytyltransferase activity, whereas olive MPBQ MT belongs to the *PLNO2490* superfamily, with MPBQ methyltransferase activity. In addition, Pfam analysis indicated the presence of an UbiA prenyltransferase domain between amino acids 137-396 for OepHPT, and a methyltransferase and an UbiE methyltransferase domain between amino acids 123-215 and 232-262, respectively, in the case of OepMPBQ MT.

Various distinctive conserved motifs were identified in the alignment of the HPT deduced amino acid sequences (**Figure 1A**). Among them, OepHPT contains prenyl-DP and divalent cation binding motifs typical for polyprenyltransferases (Lopez et al., 1996; Collakova and DellaPenna, 2001). In addition, an Asp-rich motif is also present in the olive HPT sequence, which is involved in substrate binding (Huang et al., 2014). Regarding olive MPBQ MT (**Figure 1B**), three conserved domains (SAM I, II, and III) were detected in the alignment, which are characteristic of *S*-adenosylmethionine-dependent methyltransferases (Kagan and Clarke, 1994; Joshi and Chiang, 1998). SAM I and II correspond to the binding sites for substrate *S*-adenosylmethionine, while SAM III corresponds to the binding site of catalytic products.

Regarding the subcellular localization, analysis of the deduced olive HPT and MPBQ MT protein sequences with subcellular localization prediction software such as ProtComp, WoLF PSORT, or TargetP suggests that both proteins could be located in the chloroplast. In addition, an N-terminal transit peptide with the characteristic features of chloroplast targeting peptides was detected by ChloroP software in both sequences (**Figure 1**), with a predicted cleavage site after Cys at residue 39 and Ser at residue 65 for OepHPT and OepMPBQ MT, respectively. In line with these observations, experimental evidence of chloroplast localization for HPT and MPBQ MT from sweet potato in tobacco leaves has been reported (Ji et al., 2016).

As shown in **Supplementary Figure 1**, both olive HPT and MPBQ MT proteins are highly hydrophobic based on hydropathy plotting (Kyte and Doolittle, 1982), indicating that they are membrane proteins. Concerning the membrane topology, transmembrane predictions based on a hidden Markov model (TMHMM) analysis of the olive HPT and MPBQ MT proteins were generated (**Supplementary Figure 2**). OepHPT showed nine putative transmembrane domains as reported for other plant HPT proteins (Wunnakup et al., 2018), whereas, in the OepMPBQ MT sequence, only one transmembrane domain was found in the C-terminal region at positions 316-335. This transmembrane domain was previously identified in the Arabidopsis MPBQ MT and it has been suggested that the protein is anchored to the inner chloroplast envelope membrane at this position (Motohashi et al., 2003).

**Figure 2:**
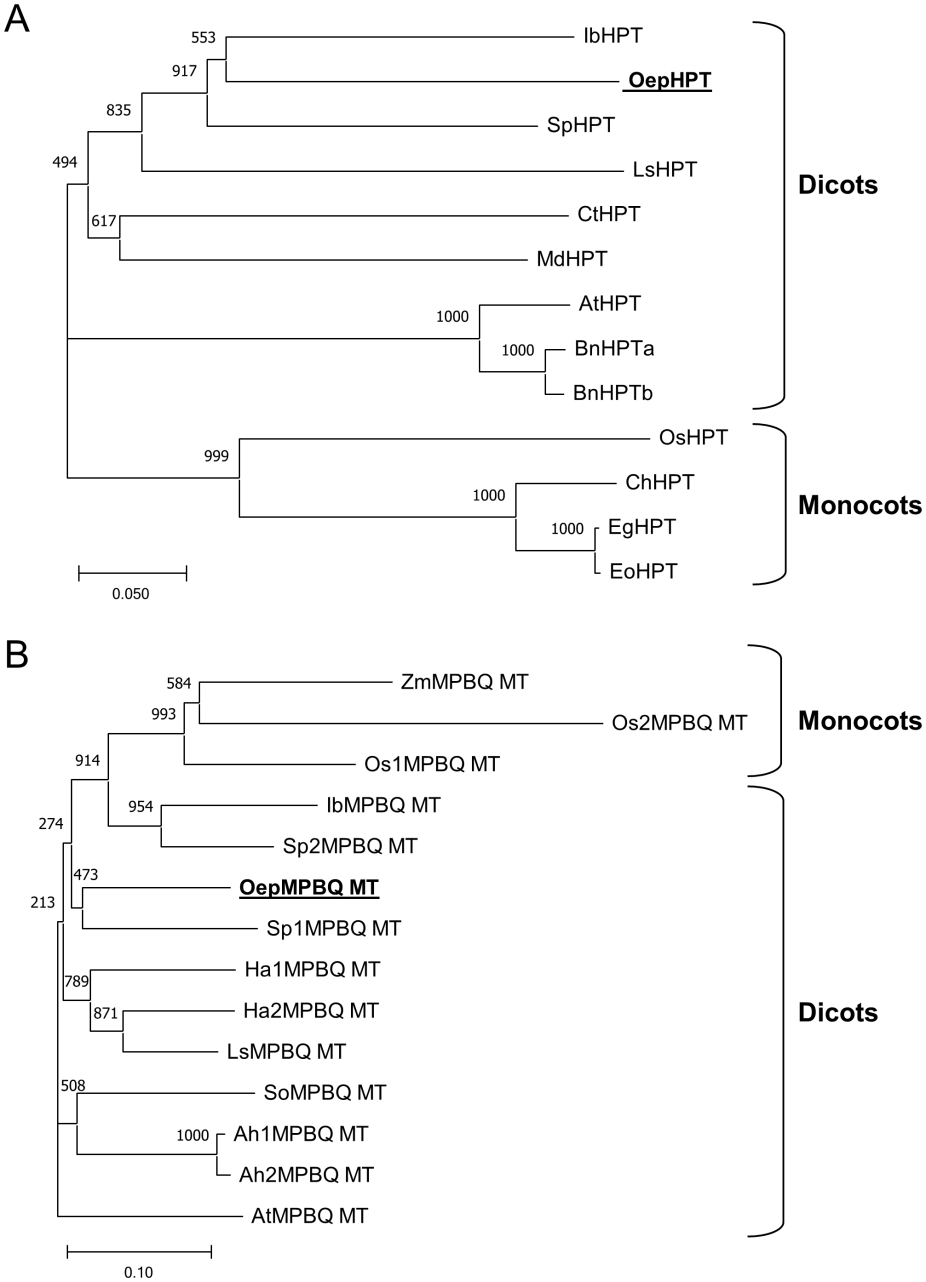
Phylogenetic tree analysis of plant homogentisate phytyltransferases **(A)** and methylphytylbenzoquinol methyltransferases **(B)**. Alignments were calculated with ClustalX and the analysis was performed using the neighbor-joining method implemented in the Phylip package using Kimura’s correction for multiple substitutions, and a 1000 bootstrap data set. TreeView was used to display the tree. Positions of the olive HPT and MPBQ MT are in bold and underlined. Accession numbers of the different HPT and MPBQ MT included in this analysis are indicated in **Supplementary Table 2.**.

Two unrooted phylogenetic trees based on deduced amino acid sequences of known and characterized plant HPT (**Figure 2A**) and MPBQ MT (**Figure 2B**) were generated to investigate the phylogenetic relationship of olive HPT and MPBQ MT, respectively. In agreement with previous findings (Chaudhary and Khurana, 2009; Ji et al., 2016), plant HPT and MPBQ MT could be classified into two separate monocot and dicot-specific clades, with OepHPT and OepMPBQ MT being positioned, respectively, in the branch accompanied by other HPT and MPBQ MT from dicots plants. It seems that HPT and MPBQ MT evolved differently between dicots and monocots species.

Altogether, sequence analysis of the olive HPT and MPBQ MT displayed the distinguishing characteristics typical of the HPT and MPBQ MT families and along with phylogenetic analysis indicated that they code for homogentisate phytyltransferase and methylphytylbenzoquinol methyltransferase enzymes, respectively.

### Tissue specificity of olive homogentisate phytyltransferase and methylphytylbenzoquinol methyltransferase genes

**Figure 3:**
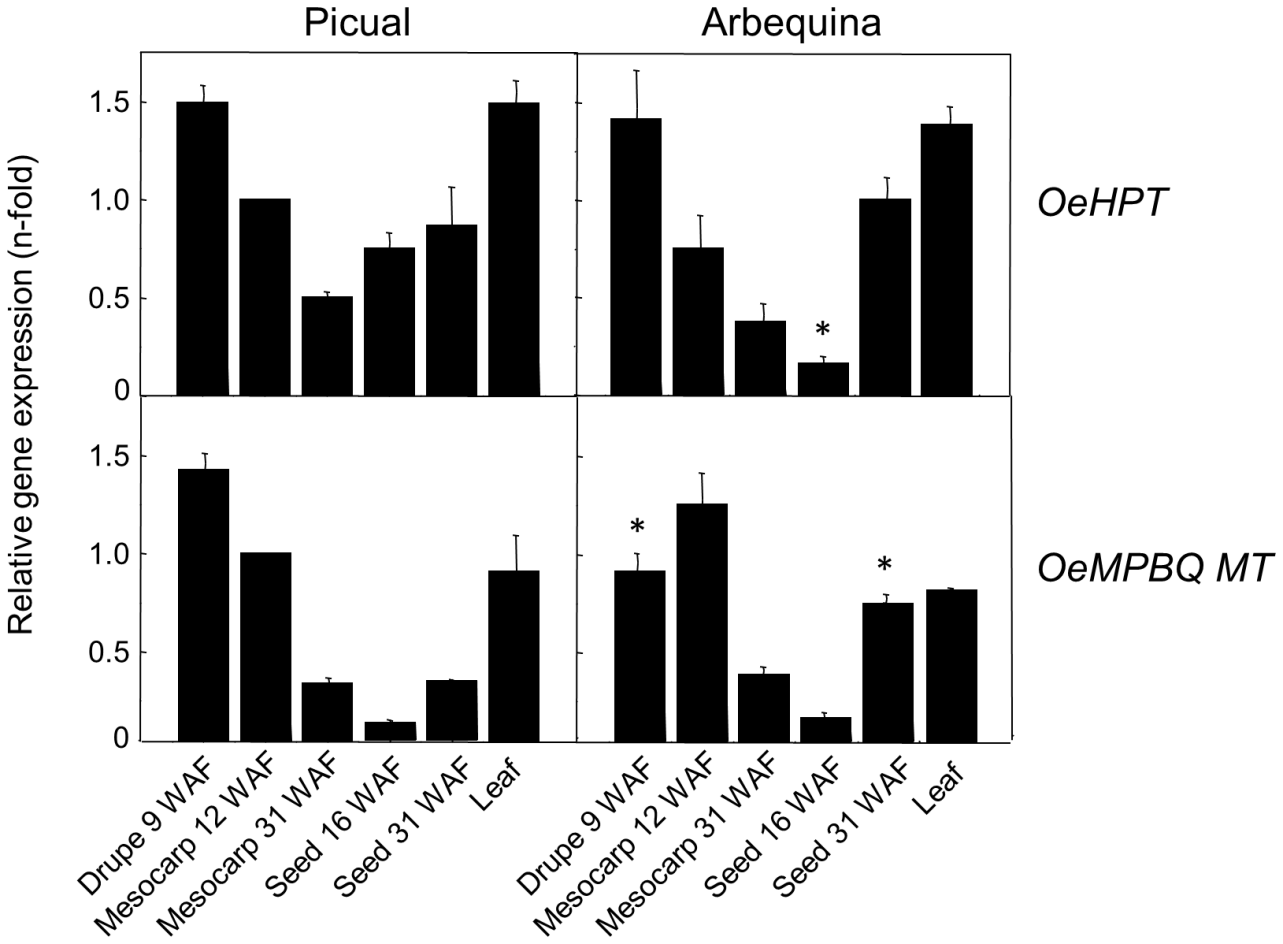
Relative expression levels of olive *HPT* and *MPBQ MT* genes in different organs and tissues of Picual and Arbequina cultivars. The relative expression levels were determined by qRT-PCR in the indicated organs and tissues as described under section “Materials and Methods”. Data are presented as means ± SD of three biological replicates. *Indicates significantly different (*P*<0.05) to ‘Picual’ by two-way analysis of variance (ANOVA) with a Bonferroni posttest in ‘Arbequina’ tissues.

Olive *HPT* and *MPBQ MT* transcript levels were determined in distinct olive organs and tissues from ‘Picual’ and ‘Arbequina’, the two main cultivars for oil production, using qRT-PCR (**Figure 3**) to investigate their physiological function. Both genes exhibit higher expression levels in green tissues from both cultivars such as young drupes, green mesocarp, and leaves compared to mature mesocarp or young seeds. Analogous observations have been reported in the case of oil palm *HPT* (Ling et al., 2016) and tomato *MPBQ MT* (Quadrana et al., 2014). The high transcript levels detected for both genes in green tissues are consistent with the elevated tocopherol content characteristic of photosynthetic organs, given the protective role of tocopherols against photooxidation and photoinactivation (Havaux et al., 2005). In addition, all these data indicate a spatial regulation of *HPT* and *MPBQ MT* genes in olive considering that they were differentially expressed in all organs and tissues studied.

### Developmental expression of homogentisate phytyltransferase and methylphytylbenzoquinol methyltransferase genes in the olive fruit in relation to the tocopherol content

Afterward, the expression levels of olive *HPT* and *MPBQ MT* genes in the olive fruit were studied in more detail. Specifically, seeds and mesocarp tissue during the development and ripening of olive fruit from the cultivars Picual and Arbequina were analyzed.

Concerning developing seeds (**Supplementary Figure 3**), gene expression analysis revealed that *HPT* and *MPBQ MT* genes exhibited an increase in their transcript levels during the whole period of olive fruit development and ripening in both cultivars, showing maximum values for both genes at 35 and 31 WAF for ‘Picual’ and ‘Arbequina’, respectively. The detected increase of *HPT* and *MPBQ MT* transcripts during seed development has been previously observed in other plants, as in the case of the rice and blackberry *HPT* (Wang et al., 2013; Yang et al., 2020), and the *HPT* and *MPBQ MT* from oat (Gutierrez-Gonzalez and Garvin, 2016). However, in the case of olive, this increase in *HPT* and *MPBQ MT* expression levels detected in the seed is not in accordance with the slight continuous decrease of tocopherol content observed in oils from Picual and Arbequina cultivars obtained at different stages of development and ripening (Pérez et al., 2019), likely because the contribution of the seed to the final composition of the VOO is very minor (Hernández et al., 2016).

**Figure 4:**
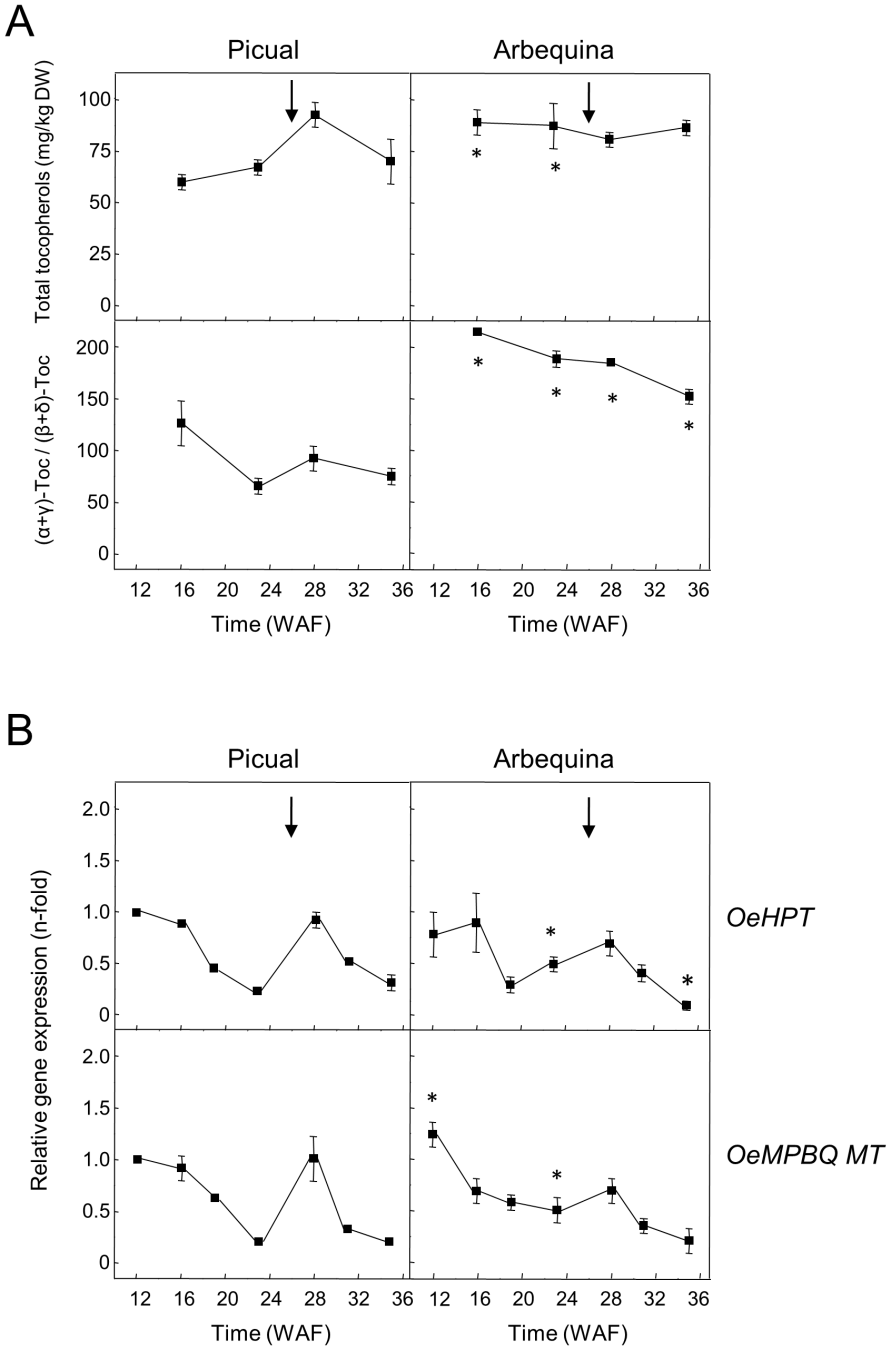
Total tocopherol content and (α+γ)-tocopherol/(β+δ)-tocopherol ratio **(A)**, and relative expression levels of olive *HPT* and *MPBQ MT* genes **(B)** in the mesocarp tissue from Picual and Arbequina cultivars. The beginning of fruit ripening corresponding to the appearance of a pink-purple colour is denoted by an arrow. At the indicated stages, tocopherols were analyzed by HPLC, and the relative expression levels were determined by qRT-PCR as described under section “Materials and Methods”. Data are presented as means ± SD of three biological replicates. *Indicates significantly different (*P*<0.05) to ‘Picual’ by two-way ANOVA with a Bonferroni posttest in ‘Arbequina’.

A similar investigation was performed in the olive mesocarp of Picual and Arbequina cultivars (**Figure 4**). The observed pattern in *HPT* and *MPBQ MT* expression levels during olive mesocarp development and ripening (**Figure 4B**) is in line with that of the total tocopherol content and the (α+γ)-tocopherol/(β+δ)-tocopherol ratio (**Figure 4A**), respectively, with a peak at 28 WAF in the case of ‘Picual’, coinciding with the beginning of the ripening period. In the case of the cultivar Koroneiki, a decrease in their expression levels has been described for both genes (Georgiadou et al., 2015). All these data point out that in olive fruit the expression of *HPT* and *MPBQ MT* genes seems to be temporally regulated.

Concerning other fruits, a reduction of *HPT* expression levels has been reported during fruit ripening in apple and the pulp of *Citrus* species (Seo et al., 2011; Rey et al., 2021b), whereas a significant up-regulation was observed for oil palm *HPT* at late stages of mesocarp ripening (Ling et al., 2016). On the contrary, *MPBQ MT* from tomato fruit and the pulp of *Citrus* species exhibited comparable transcript levels along development and ripening (Quadrana et al., 2014; Rey et al., 2021b).

**Figure 5:**
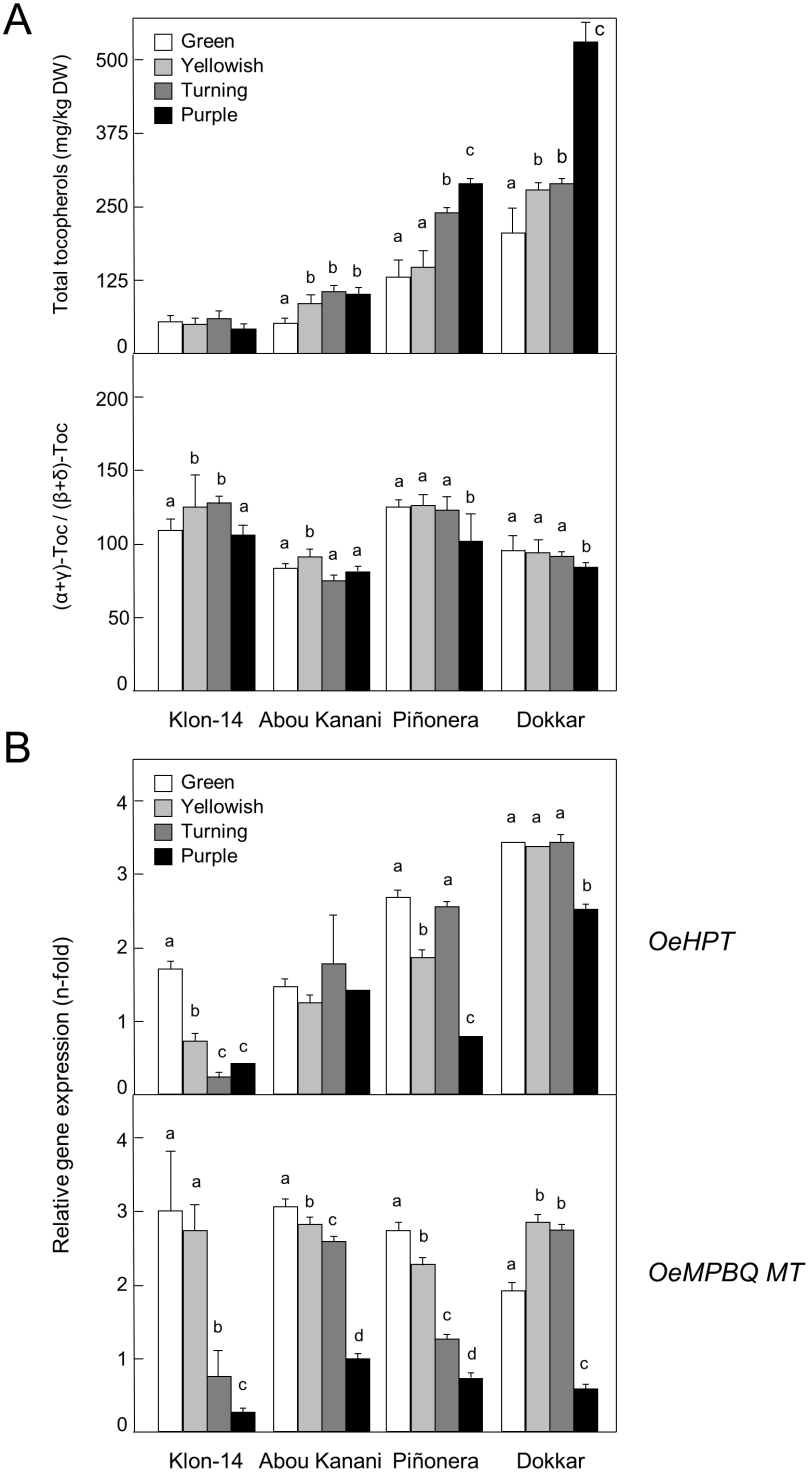
Total tocopherol content and (α+γ)-tocopherol/(β+δ)-tocopherol ratio **(A)**, and relative expression levels of olive *HPT* and *MPBQ MT* genes **(B)** in the mesocarp tissue from different olive cultivars. At the indicated stages, tocopherols were analyzed by HPLC, and the relative expression levels were determined by qRT-PCR as described under section “Materials and Methods”. Data are presented as means ± SD of three biological replicates. Different letters denote significant differences (*P* < 0.05) for each gene and cultivar by one-way ANOVA followed by Tukey’s post-test for multiple comparisons.

Our study was expanded to other olive cultivars characterized by a low (190 and 285 mg/kg oil for ‘Klon-14’ and ‘Abou Kanani’, respectively) or high (820 and 1610 mg/kg oil for ‘Piñonera’ and ‘Dokkar’, respectively) tocopherol content in their oils (Pérez et al., 2019), using mesocarp tissue corresponding to four different representative stages of fruit development and ripening (**Figure 5**). Unlike *OeMPBQ MT* which shows similar expression levels for each stage of these four cultivars (**Figure 5B**), the transcript levels of *OeHPT* were higher in those cultivars with a higher tocopherol content such as ‘Piñonera’ and ‘Dokkar’ (**Figure 5A**). In contrast, in ‘Klon-14’ and ‘Abou Kanani’, characterized by a low content of tocopherols, their expression levels were lower. These data suggest that HPT, but not MPBQ MT, could be involved in the regulation of the tocopherol biosynthetic pathway in olive mesocarp at the transcriptional level, which is consistent with its role in catalyzing the first committed step of this route. However, the variation in the *OeHPT* and *OeMPBQ MT* transcript levels during olive mesocarp development and ripening (**Figure 5B**) was not parallel to that of the total tocopherol content and the (α+γ)-tocopherol/(β+δ)-tocopherol ratio (**Figure 5A**), respectively. This fact indicates that not only HPT but also other factors such as the availability of HGA and PDP as precursors regulate the flux of the tocopherol biosynthetic pathway (Pellaud and Mène-Saffrané, 2017).

Collectively, these results point out that olive *HPT* and *MPBQ MT* participate in the biosynthesis of the tocopherols present in VOO and their gene expression levels are cultivar-dependent, as reported from five Greek cultivars (Georgiadou et al., 2019).

### Effect of regulated deficit irrigation on homogentisate phytyltransferase and methylphytylbenzoquinol methyltransferase gene expression in the olive fruit mesocarp

**Figure 6:**
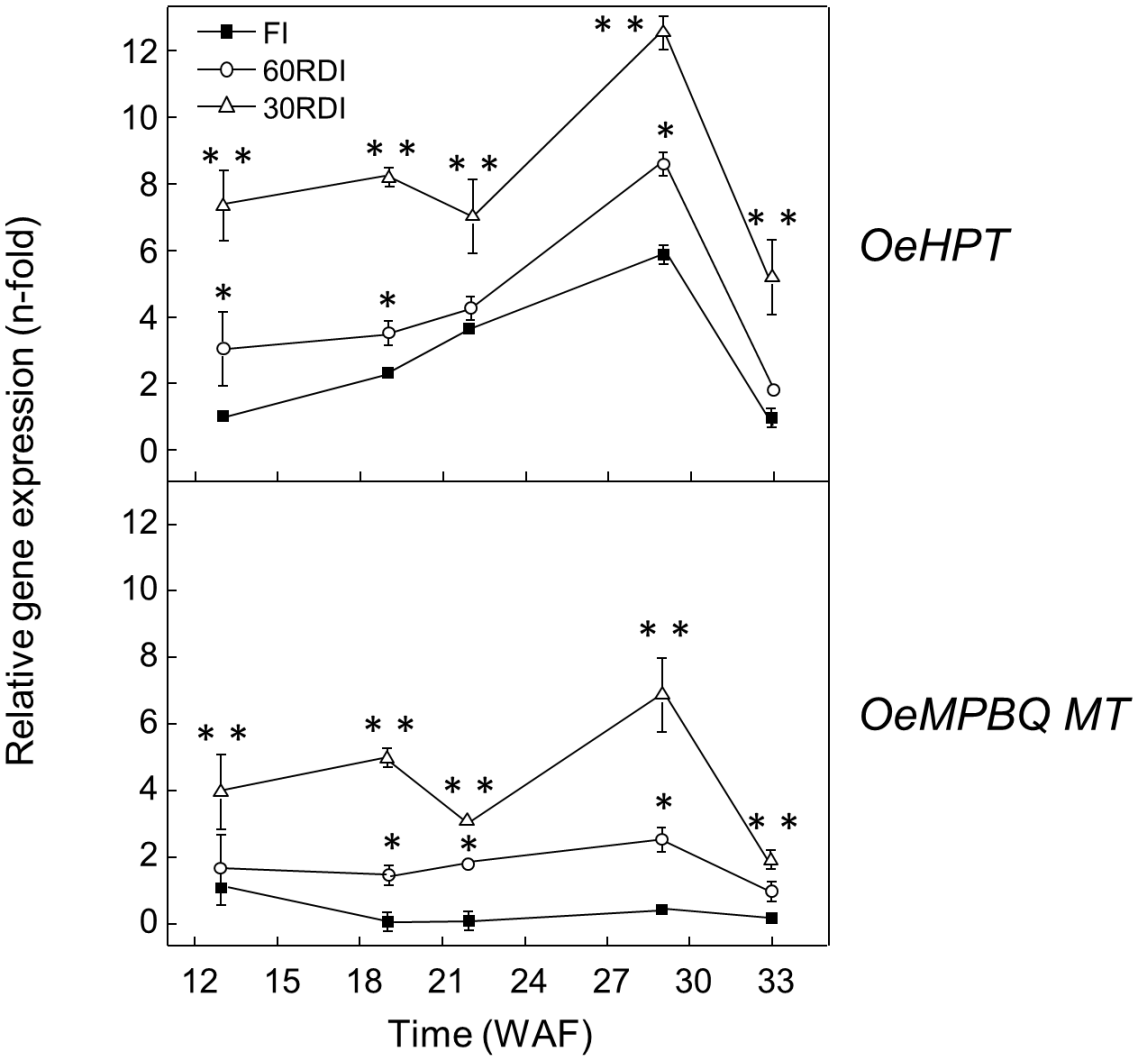
Effect of regulated deficit irrigation (RDI) treatments on the relative expression levels of olive *HPT* and *MPBQ MT* genes in the mesocarp tissue from cultivar Arbequina during olive fruit development and ripening. The relative expression levels were determined by qRT-PCR at the indicated stages of fruit development as described in “Materials and Methods”, using the expression level of the corresponding gene at 13 WAF from full irrigation (FI) treatment as calibrator. Data are presented as means ± SD of three biological replicates. *Indicates that 60 RDI is significantly different (*P* < 0.05) to FI by two-way ANOVA with a Bonferroni post-test. **Indicates that 30 RDI is significantly different (*P* < 0.05) to FI by two-way ANOVA with a Bonferroni post-test.

It is well described that drought stress increases tocopherol content in the leaves of several plant species, including olive juvenile trees (Baccari et al., 2020). The effect of three distinct RDI treatments on the oil accumulation, fatty acid profile, and fatty acid desaturase gene expression levels in ‘Arbequina’ fruit mesocarp has been previously studied by our group (Hernández et al., 2018). However, data related to the effect of water stress on the expression levels of olive genes belonging to the tocopherol biosynthetic pathway are lacking. In the present work, higher expression levels of *HPT* and *MPBQ MT* genes were found along all the fruit developmental and ripening processes in ‘Arbequina’ mesocarp from olives subjected to 30RDI and 60RDI treatments, which produced substantial levels of water stress, in comparison to FI treatment (**Figure 6**). This result is in agreement with the increase of tocopherol content reported by García et al. (2017) in oils obtained from the same olive fruit samples when comparing FI and RDI treatments (255, 327, and 321 mg/kg oil; for FI, 60RDI and 30RDI treatments, respectively). Therefore, the tocopherol biosynthetic pathway appears to be transcriptionally up-regulated in water-stressed olive mesocarp, with *OeHPT* and *OeMPBQ MT* genes increasing their expression levels and causing an increment in the tocopherol content in ‘Arbequina’ mesocarp and, consequently, in the corresponding oils. In line with these data, the transcription of genes involved in tocopherol biosynthesis in olives from the Koroneiki cultivar was up-regulated in response to lower rainfall during consecutive seasons (Georgiadou et al., 2016).

An increase in the *HPT* expression levels has also been detected in leaves from other plants under drought stress, such as *Cistus cetricus* (Munné-Bosch et al., 2009), lettuce (Ren et al., 2011), sweet potato (Ji et al., 2016), or barley (Schuy et al., 2019). Furthermore, it has been reported that drought-induced expression of *HPT* from *Solanum chilense* in tobacco plants results in the accumulation of α-tocopherol and increases tolerance to drought stress by delaying foliar tissue damage (Espinoza et al., 2013), demonstrating the role of *HPT* in drought stress resistance. On the contrary, no significant effect on *MPBQ MT* transcript levels has been described in plants under drought stress to date.

### Transcriptional regulation of homogentisate phytyltransferase and methylphytylbenzoquinol methyltransferase genes in the olive fruit mesocarp under different environmental stresses

To investigate the effect of several abiotic stresses on the expression levels of the olive *HPT* and *MPBQ MT* genes in mesocarp tissue from ‘Picual’ and ‘Arbequina’, olive tree branches holding olive fruit at the turning stage (28 WAF) were incubated for 24 h modifying the standard conditions (25°C with 12 h light/12 h dark cycle) dependent on the effect to be examined. No significant changes in the olive *HPT* and *MPBQ MT* transcript levels were observed in the mesocarp tissue when olive fruit were incubated under the above-mentioned standard conditions (**Supplementary Figure 4**).

**Figure 7:**
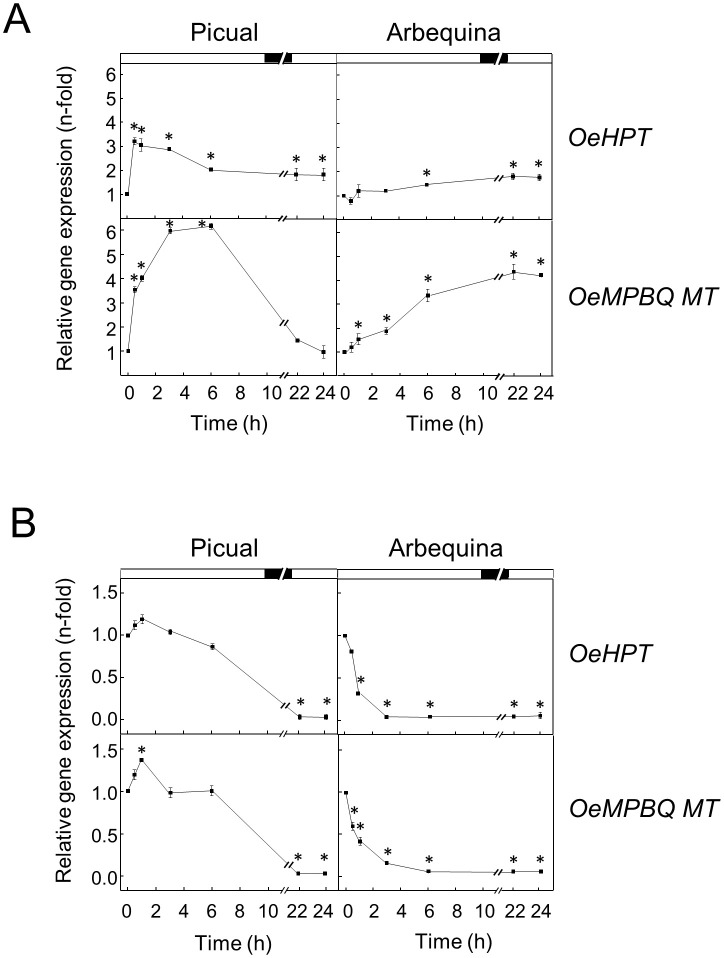
Effect of low **(A)** and high **(B)** temperature on the relative expression levels of olive *HPT* and *MPBQ MT* genes in the mesocarp tissue from Picual and Arbequina cultivars. Olive tree branches with about 100 olive fruits (28 WAF) were incubated using standard conditions except that the temperature was 15°C **(A)** or 35°C **(B)**. At the indicated times, the relative expression levels were determined by qRT-PCR as described in “Materials and Methods”, using the expression level of the corresponding gene at zero time as a calibrator. Data are presented as means ± SD of three biological replicates. *Indicates significantly different (*P*<0.05) to time 0 h by two-way ANOVA with a Bonferroni posttest. Boxes in the upper part indicate light (open) or dark (closed) periods.

It has been shown that tocopherols play a key role in the low-temperature adaptation of plants such as maize (Leipner et al., 1999) or Arabidopsis (Maeda et al., 2008). When olive fruit was incubated at a low temperature (15°C) a significant increment in the expression levels of *HPT* and *MPBQ MT* genes in the mesocarp from both cultivars was noticed (**Figure 7A**). In ‘Picual’, a strong transient increase was observed for the transcript levels of both genes with maximum values after 0.5-1 h and 3-6 h of treatment for *HPT* and *MPBQ MT*, respectively. In contrast, in ‘Arbequina’ mesocarp a continuous increase was detected along all the incubation time, being higher in the case of the *OeMPBQ MT* gene. A similar up-regulation of *HPT* and *MPBQ MT* transcript levels has been described in mandarin fruit and grapefruit during long-term cold storage (Rey et al., 2021a; Rey et al., 2021c). In the same way, when *Chlamydomonas reinhardtii* cells were incubated at low temperature, an increase in *HPT* expression levels was observed (Gálvez-Valdivieso et al., 2015). In addition, HPT has been demonstrated to be essential for cold tolerance and low-temperature adaptation, since Arabidopsis and rice *vte2* mutants were hypersensitive to cold stress (Maeda et al., 2006; Wang et al., 2017; Zhang et al., 2018).

Regarding high temperature, when olive fruit from both cultivars were incubated at 35°C the transcript levels of *HPT* and *MPBQ MT* showed a severe reduction until undetectable values were reached (**Figure 7B**). Both genes started to decrease their transcript levels after 6 h of incubation in the case of ‘Picual’, while in ‘Arbequina’ the diminution began sharp and rapidly from the start of the treatment. Interestingly, it has been reported that total tocopherol concentrations in olive fruit at the end of the oil accumulation period were generally higher when the air temperature was increased by 4°C in young trees from Arbequina and Coratina cultivars (Hamze et al., 2022). However, these authors suggest that the observed increase in total tocopherols appeared to be related to a reduction in fruit oil concentration with heating. In line with our results, *C. reinhardtii* cells incubated at high temperature exhibited a slight decrease in *HPT* expression levels (Gálvez-Valdivieso et al., 2015), and leaves of *Cinnamomum camphora* under high temperature showed a down-regulation of the *MPBQ MT* transcript levels (Wang et al., 2023).

**Figure 8:**
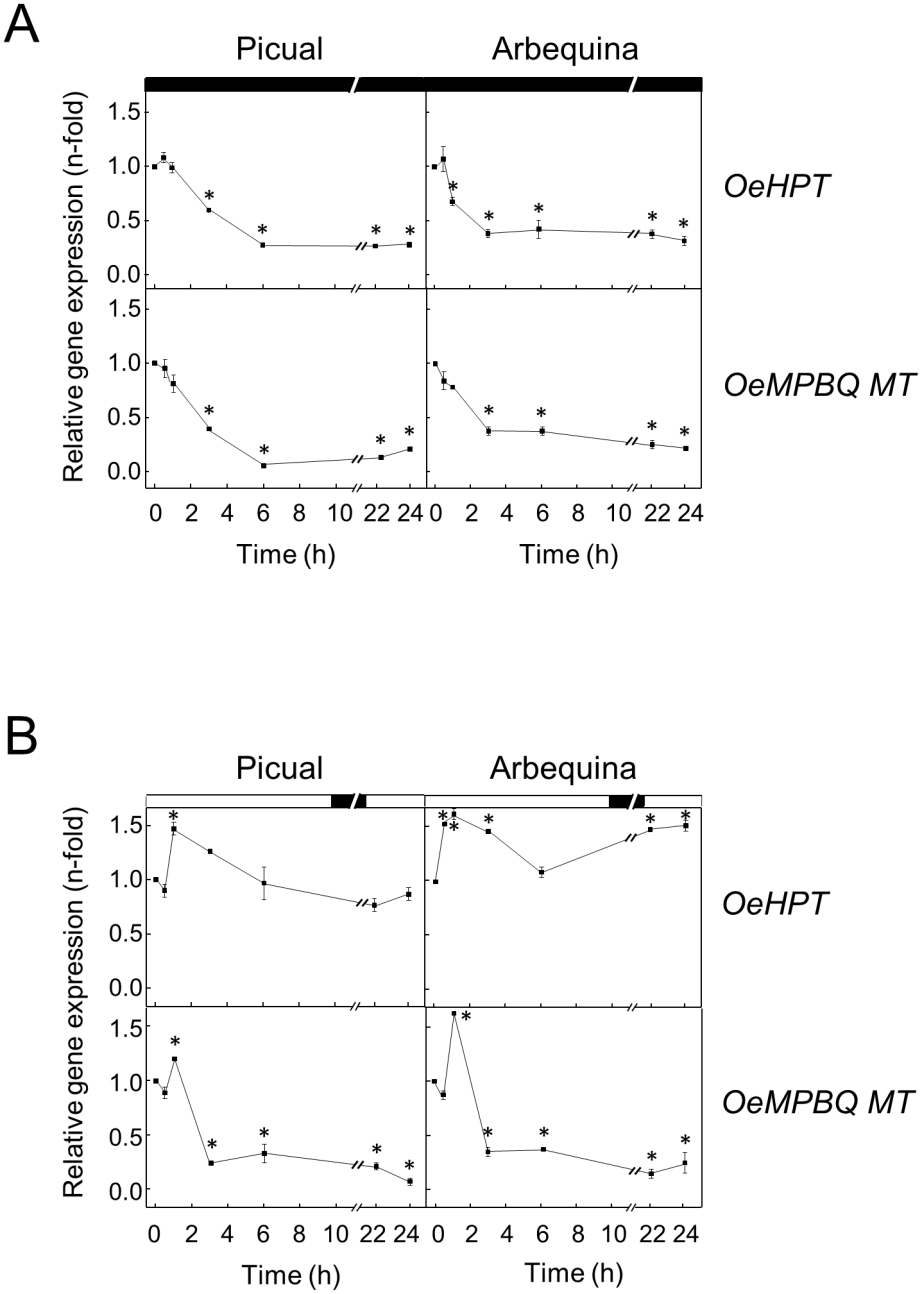
Effect of darkness **(A)** and wounding **(B)** on the relative expression levels of olive *HPT* and *MPBQ MT* genes in the mesocarp tissue from Picual and Arbequina cultivars. Olive tree branches with about 100 olive fruits (28 WAF) were incubated using standard conditions except that the olive fruit were incubated under darkness **(A)** or were mechanically damaged **(B)**. At the indicated times, the relative expression levels were determined by qRT-PCR as described in “Materials and Methods”, using the expression level of the corresponding gene at zero time as a calibrator. Data are presented as means ± SD of three biological replicates. *Indicates significantly different (*P*<0.05) to time 0 h by two-way ANOVA with a Bonferroni posttest. Boxes in the upper part indicate light (open) or dark (closed) periods.

Previous studies in plant tissues demonstrated that the tocopherol content is light regulated (Lichtenthaler, 2007). To examine whether darkness affects *HPT* and *MPBQ MT* expression levels in olive mesocarp from Picual and Arbequina cultivars, olive branches were incubated at 25°C for 24 h in the darkness. Expression analysis showed a substantial decline of *OeHPT and OeMPBQ MT* transcript levels in both cultivars mostly during the first 3-6 h of treatment, remaining with small values the rest of the experiment (**Figure 8A**). In agreement with these results, a down-regulation of the *HPT* expression levels has also been observed in dark-grown tomato fruit and grapefruit (Gramegna et al., 2019; Rey et al., 2021c). In addition, Gálvez-Valdivieso et al. (2015) reported a fast and strong reduction of *HPT* transcripts in *C. reinhardtii* cells transferred to darkness. In the case of *MPBQ MT*, very low transcript levels were found in the leaves of Arabidopsis plants grown in the dark (Motohashi et al., 2003). These data indicate that *HPT* and *MPBQ MT* are regulated by light.

Finally, the impact of wounding on the *HPT* and *MPBQ MT* gene expression levels was investigated for the first time. In particular, this effect was studied in the mesocarp of olive fruit from Picual and Arbequina cultivars subjected to mechanical damage from branches incubated at standard conditions. In both cultivars, *OeHPT* and *OeMPBQ MT* transcript levels exhibited a slight transient induction, showing peak values after 1 h of treatment and followed by a decrease, especially marked in the case of *MPBQ MT* (**Figure 8B**). Notably, a role for tocopherol biosynthesis in Arabidopsis basal immunity to bacterial infection has been recently proposed (Stahl et al., 2019). Specifically, a substantial increase of *HPT* gene expression levels was detected in Arabidopsis leaf in response to inoculation with *Pseudomonas syringae*, and an enhanced susceptibility toward *P. syringae* was observed in Arabidopsis *vte2* mutant plants. In contrast, no significant differences in *HPT* and *MPBQ MT* transcript levels were found in sweet potato leaves infected with the bacterial pathogen *Pectobacterium chrysanthemi* compared to the control (Ji et al., 2016).

## Conclusion

The isolation and characterization of olive *HPT* and *MPBQ MT* genes have been performed. Sequence analysis of both genes shows that they code for HPT and MPBQ MT enzymes, respectively. Tocopherol and expression analysis reveals not only a spatial and temporal regulation of olive *HPT* and *MPBQ MT* transcript levels in olive fruit in the course of development and ripening but also indicates that both genes are involved in the biosynthesis of the tocopherols present in VOO. These data also point out that olive *HPT* and *MPBQ MT* gene expression is cultivar-dependent and suggest that in olive mesocarp HPT, but not MPBQ MT, could be involved in the regulation of the tocopherol biosynthetic pathway at the transcriptional level. Our results have also shown that the expression of *HPT* and *MPBQ MT* genes in olive fruit is regulated by water status, temperature, light, and wounding, suggesting that HPT and MPBQ MT participate in the response to abiotic stresses. This study represents substantial progress in the knowledge of the regulation of tocopherol biosynthesis in olive fruit. In addition, it will help to establish optimum conditions for olive tree cultivation and olive fruit harvesting to obtain VOO with enhanced α-tocopherol content. Furthermore, this information will allow the generation of molecular markers for the marker-assisted selection of new olive cultivars with increased tocopherol content in the VOO.

## Data Availability

The datasets presented in this study can be found in online repositories. The names of the repository/repositories and accession number(s) can be found below: https://www.ncbi.nlm.nih.gov/genbank/, PQ479102 https://www.ncbi.nlm.nih.gov/genbank/, PQ479103.
