# Supplementary material for: Molecular characterization of homogentisate phytyltransferase and methylphytylbenzoquinol methyltransferase genes from olive fruit with regard to the tocopherol content and the response to abiotic stresses
Source: Front Plant Sci. 2025 Mar 3;16:1526815. doi: 10.3389/fpls.2025.1526815 (PMC11911349; doi:10.3389/fpls.2025.1526815)
Supplement: Supplementary file 1 [file DataSheet1.pdf]

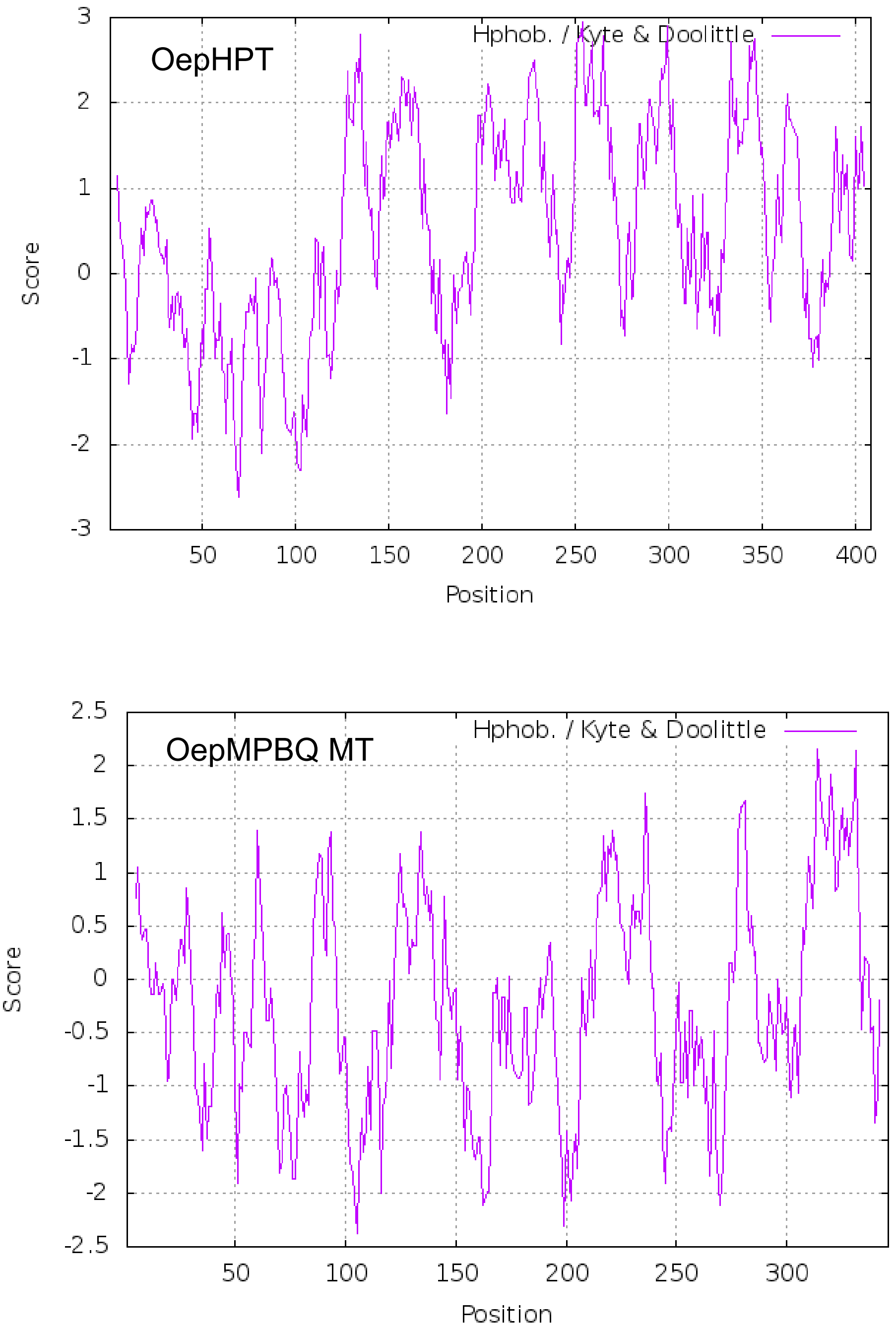

Supplementary Figure 1. Hidropathy plots for olive HPT and MPBQ MT sequences obtained by the method of Kyte and Doolittle (1982).

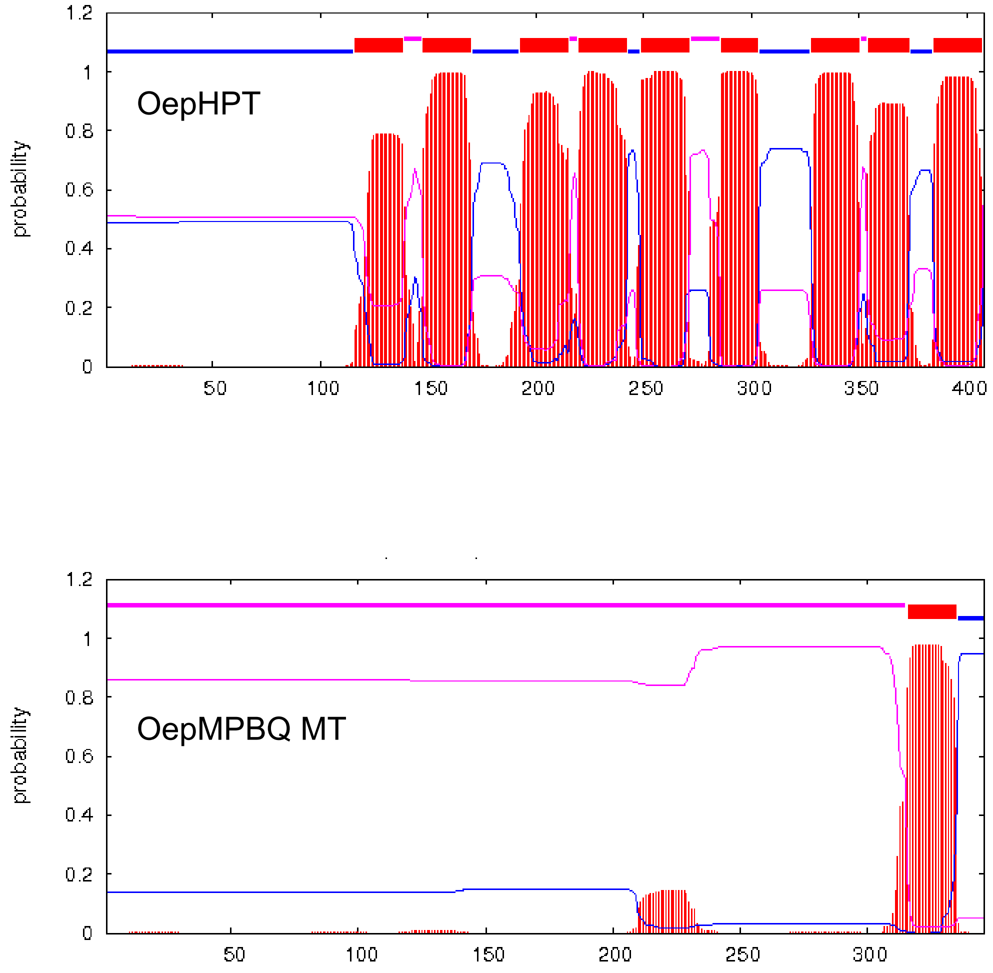

Supplementary Figure 2. Predicted transmembrane domains shown in red for olive HPT and MPBQ MT sequences identified by TMHMM analysis.

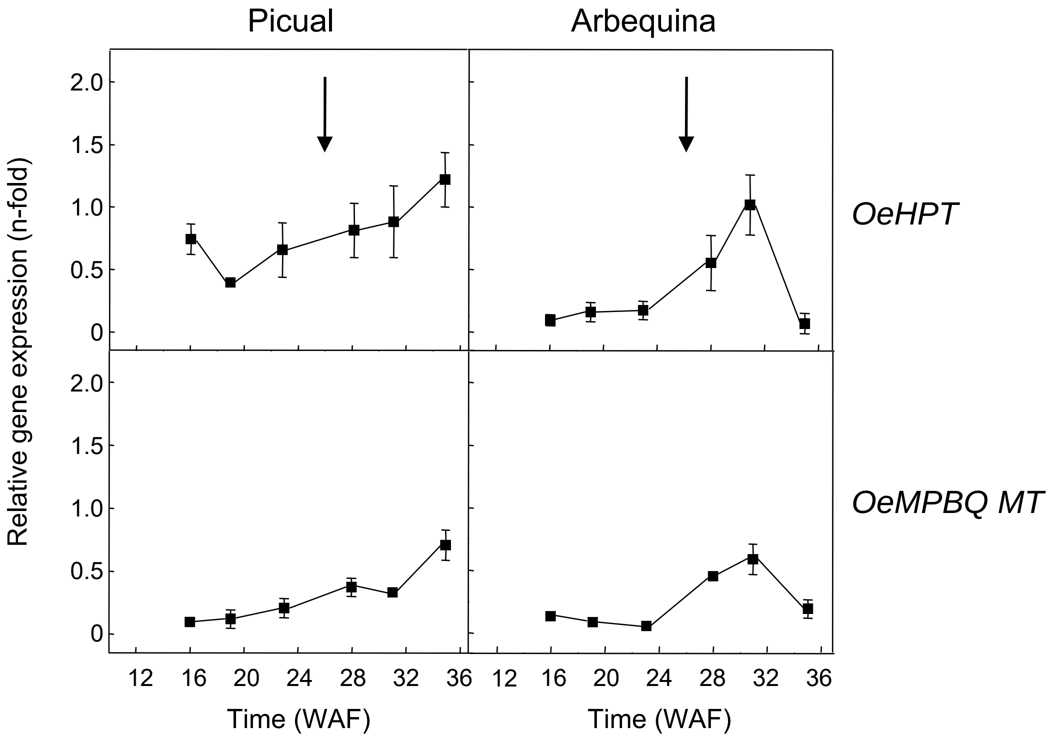

Supplementary Figure 3. Relative expression levels of olive *HPT* and *MPBQ MT* genes in the seed tissue from Picual and Arbequina cultivars. The beginning of fruit ripening corresponding to the appearance of pink-purple color is denoted by an arrow. The relative expression levels were determined by qRT-PCR at the indicated stages of fruit development as described under Materials and methods. Data are presented as means ± SD of three biological replicates.

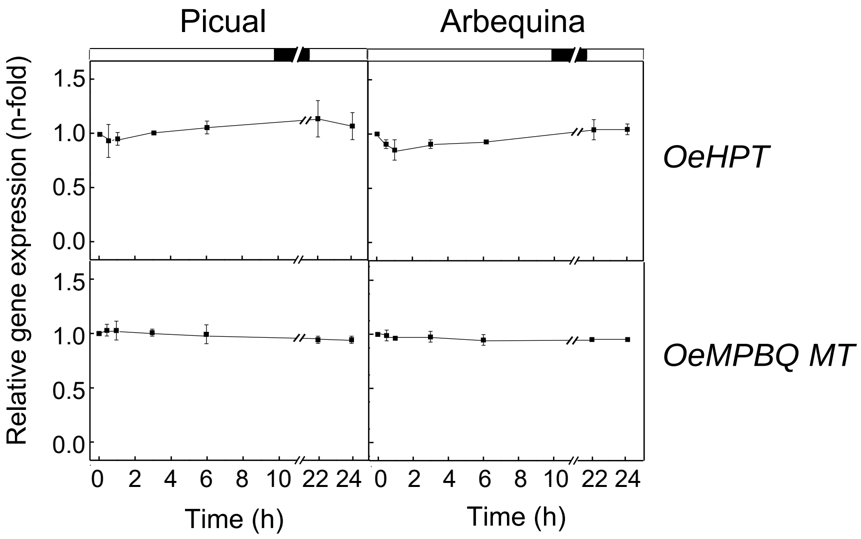

Supplementary Figure 4. Effect of incubation under the standard conditions on the relative expression levels of olive *HPT and MPBQ MT* genes in the mesocarp tissue from Picual and Arbequina cultivars. Branches with about 100 olive fruit (28 WAF) were incubated using standard conditions. At the indicated times, relative expressions levels were determined by qRT-PCR as described in Materials and methods, using the expression level of the corresponding gene at zero time as calibrator. Data are presented as means ± SD of three biological replicates. Boxes in the upper part indicate light (open) or dark (closed) periods.
