# Supplementary material for: Molecular characterization of homogentisate phytyltransferase and methylphytylbenzoquinol methyltransferase genes from olive fruit with regard to the tocopherol content and the response to abiotic stresses
Source: Front Plant Sci. 2025 Mar 3;16:1526815. doi: 10.3389/fpls.2025.1526815 (PMC11911349; doi:10.3389/fpls.2025.1526815)
Supplement: Supplementary file 2 [file Table1.docx]

Table S1. Sequences of the primers pairs used for gene expression analysis by qRT-PCR in the present study.

| Gene | Sequence | Amplicon size (bp) |
| --- | --- | --- |
| *OepHPT* | Forward: 5’- CGCTTCTTGAGATGGCTTATC -3’ | 104 |
|  | Reverse: 5’- TGAAGCCAAAGCAATATGACC -3’ |  |
| *OepMPBQ MT* | Forward: 5’- GACTCACCTTTGCAGCTTGG -3’ | 204 |
|  | Reverse: 5’- CAGAAAGAAACGGTGGAACG -3’ |  |
| *OeUBQ2* | Forward: 5’-AATGAAGTCTGTCTCTCCTTTGG-3’ | 132 |
|  | Reverse: 5’-AAGGGAAATCCCATCAACG-3’ |  |
